# Advanced Mesodermal (Müllerian) Adenosarcoma of the Ovary: Metastases to the Lungs, Mouth, and Brain

**DOI:** 10.1155/2015/403431

**Published:** 2015-12-30

**Authors:** A. Daskalaki, S. Xenaki, E. Athanasakis, E. Chrysos, G. Chalkiadakis

**Affiliations:** Department of General Surgery, University Hospital of Heraklion, 71500 Crete, Greece

## Abstract

*Background*. A malignant mixed Müllerian tumor (MMMT) is a malignant neoplasm found in the uterus, the ovaries, the fallopian tubes, and other parts of the body that contains both carcinomatous (epithelial tissue) and sarcomatous (connective tissue) components. Outcome of MMMTs is determined primarily by depth of invasion and stage. The metastatic background of these lesions is controversial and unknown.* Case Report*. A 75-year-old woman was admitted to the hospital with anorexia, weakness, and persistent coughing. The imaging exams revealed a solid, promiscuous lesion of 16 × 14 cm in dimensions located into the small pelvis, surrounding the uterus and the ovaries. The patient underwent exploratory laparotomy. The mass was removed and the histological examination of the specimen revealed an advanced mesodermal adenocarcinoma of the ovary (MMMT). Nine days after the operation the patient presented with metastatic lesions in the mouth as well as the lungs. Within a month after the discharge from the hospital metastatic lesions of the MMMT were also depicted in the CT brain scan.* Conclusion*. Despite the fact that sarcomas have a long-term metastatic potential, to our knowledge this is the first case of Müllerian adenosarcoma presenting with such extraperitoneal metastases.

## 1. Introduction

Ovarian malignancies are very common and may develop in the form of either epithelial tumors accounting for about 85–90% of all ovarian malignancies, germ cell tumors, sex cord-stromal tumors, or mixed tumors. Single-port prophylactic laparoscopic bilateral salpingo-oophorectomy is a very promising procedure offering the benefits of minimizing the risk of ovarian cancer in patients with mutant base as well as less postoperative pain, best esthetic outcome, and faster and easier extraction of the surgical specimens. One particularly rare type of ovarian malignancies is the Müllerian adenosarcoma. To our knowledge this is the first report on such a type of malignancy, presenting with metastases to the lungs, mouth, and brain.

## 2. Case Presentation

**Figure 1:**
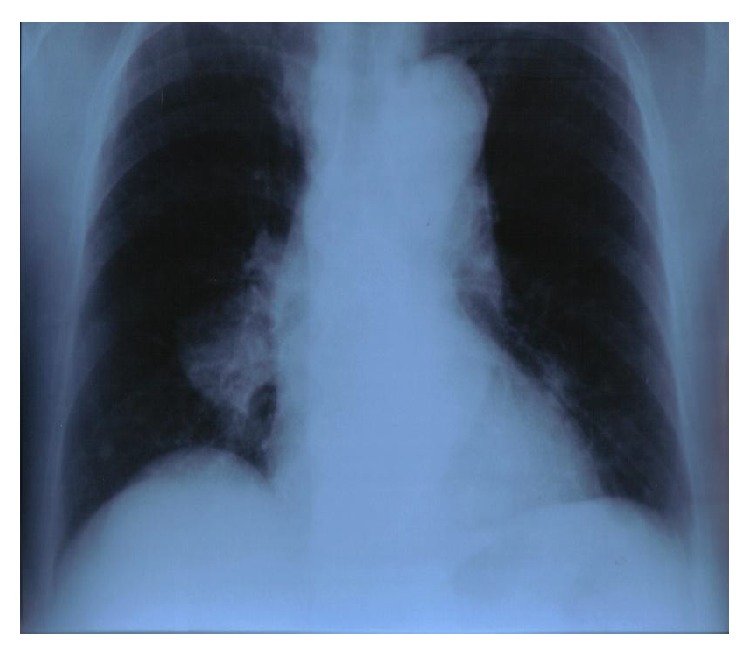
X-ray of the chest.

**Figure 2:**
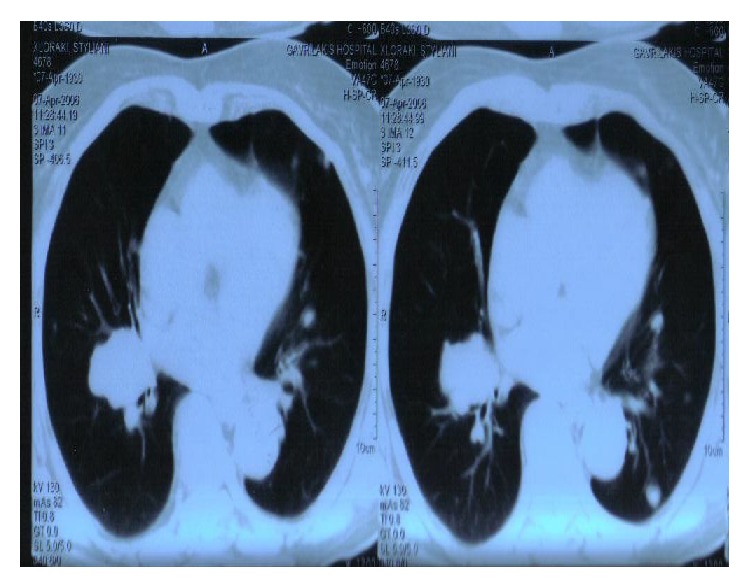
CT scan of the thorax.

**Figure 3:**
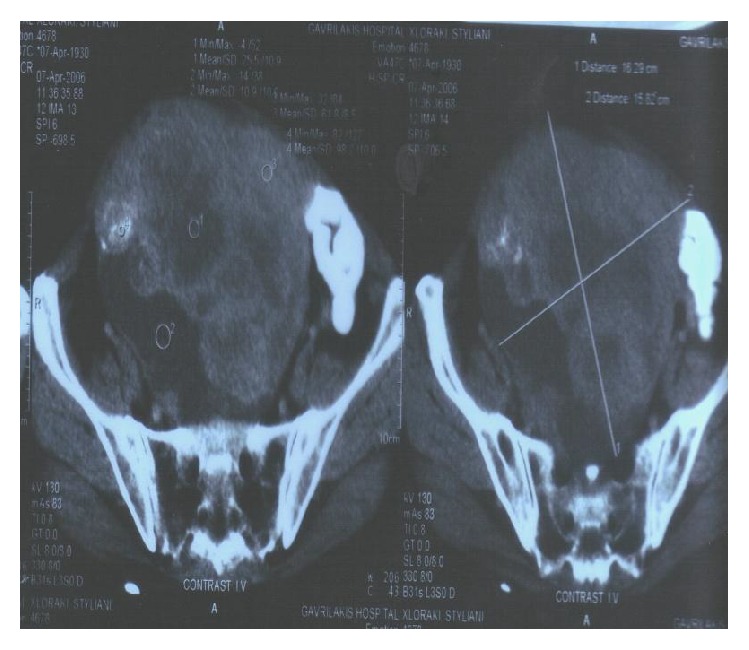
Brain CT scan.

**Figure 4:**
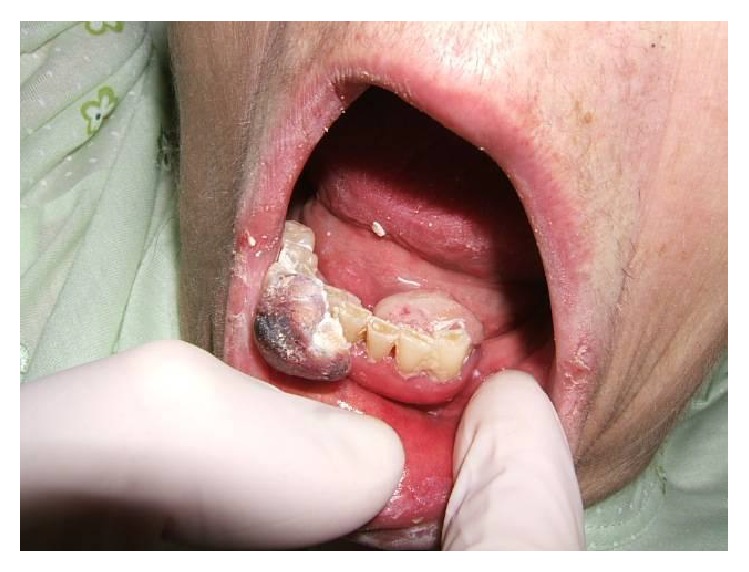
Metastasis in the oral cavity.

A 75-year-old woman was admitted to our department with a 4-day history of anorexia, weakness, and persistent coughing. She had a clear medical history without cardiac, pulmonary, or any other problems. During the clinical examination a large mass was palpated in the lower abdomen. A simple chest X-ray (Figure 1) was conducted which revealed a large, solid lesion in the median lobe of the right lung that was consistent with lung cancer. The laboratory findings were normal. A biopsy from the right bronchus was obtained by bronchoscopy and showed no malignancy as also confirmed by the bronchial cytological result. The patient was further submitted to a colonoscopy where no abnormalities were obtained. An ultrasound of the upper and lower abdomen revealed a solid, promiscuous lesion of 16 × 14 cm in dimensions located into the small pelvis, surrounding the uterus and the ovaries. The CT scan of the thorax (Figure 2) showed a 4 cm multilobed mass behind and under the hilum of the right lung and multiple nodular lesions in both lungs. The abdominal CT scan (Figure 3) confirmed the presence of the known mass and revealed no lymphadenopathy. Consequently, the patient underwent exploratory laparotomy, during which a large, encapsulated mass was found to emerge deep from the small pelvis, extend up to the umbilicus region, and infiltrate the uterus, the adnexa, and the right pelvic wall, displacing upwards and to the left the rectum and the sigmoid colon. During the surgical manipulations both ureters were inadvertently injured and surgically repaired. Appendectomy was preformed, while a glandule located at the right pelvic wall was removed. Biopsies from the surgical specimen including the appendix and the glandule were sent. The postoperative period was uneventful. On the ninth postoperative day the patient complained about two small-sized painful tumorlike lesions located in the lower jaw (Figure 4). Because of the progressive increase in their size, the lesions were removed and sent for biopsy. A few days later the results from the pathological examination of the surgical specimen were obtained and revealed an adenosarcoma of the right ovary, consisting of high-degree homologous and heterologous sarcomatous elements, expanding to the neighboring fibroid-fat tissue and too close (almost in contact) to the right ureter, part of which was incorporated into the specimen. Both the uterus and the fallopian tubes were free of malignancy. The biopsy from the glandule of the right pelvic wall showed extended infiltration from a sarcoma with morphological characters of a rhabdomyosarcoma. Finally, the appendix was free of malignancy. The patient was discharged on the 18th postoperative day. The glandules removed from the patient's mouth were found to harbor a malignant mesenchymatous neoplasm that represented a metastatic lesion from the known sarcoma. Within a month from her discharge, the patient was admitted to another hospital because of intense headache, vertigo, and vomiting. The brain CT scan showed metastases and 4 days later she passed away.

## 3. Discussion

In 1974 the term “Müllerian adenosarcoma” was used for a distinctive uterine tumor characterized by a mostly low-grade malignant stromal component and a benign, although occasionally atypical, glandular epithelial component [1]. Since that time, hundreds of similar cases have been reported in several series in the literature, most of which include a small number of patients and only one reports on up to 100 cases [2]. More than sixty histologically similar ovarian tumors have been described during the past 30 years [2–12]. In the biggest series ever to be reported [3], Eichborn et al. studied 40 patients with ovarian mesodermal adenosarcoma of grades Ia–IIIb according to FIGO classification system. They were treated with ipsilateral oophorectomy, accompanied by hysterectomy (85% of the patients), contralateral oophorectomy (65% of the patients), or no further surgical therapy (28% of the patients). The authors concluded that these tumors have a poorer prognosis than those arising from the uterus and, initially, are usually misdiagnosed. In a recent collective review [13], Mikami et al. reported on 17 cases of ovarian adenosarcoma with stage I–IIIa tumors according to FIGO classification system. The age of onset for extrauterine adenosarcoma was 48 years suggesting that hormones have a role in its etiology.

In 2010 Elnemr et al. presented a case of primary retroperitoneal Müllerian adenocarcinoma. They presented a case of a 46-year-old woman who presented with an eight-year history of lower abdominal mass. Ultrasonography (US) and computed tomography (CT) demonstrated a 15 × 10 cm cystic mass in the left lower retroperitoneum. As serial percutaneous needle aspiration cytology was negative for malignancy, she was observed for seven years. Eleven months ago, the mass was excised. The histopathology was reported as mucinous adenocarcinoma of the retroperitoneum. Six cycles of intraperitoneal (IP) chemotherapy were administered during the last six months after diagnosis of recurrence by aspiration cytology and high serum tumor markers (CEA, CA19-9). A few days ago, positron emission tomographic (PET) scanning showed evidence of local recurrence and single vertebral metastasis, so she was admitted again for systemic chemotherapy. Meticulous revision of additional sections of the tumor revealed papillary, serous, mucinous, and endometrioid subtypes of the Müllerian adenocarcinoma [14].

Quite recently in 2015 Rosas-Guerra et al. presented a case of a metastatic collision tumor in the intestine. The case was about a 71-year-old female with history of a carcinoid tumor removed 20 years before without any recurrence. The patient was admitted with intestinal occlusion symptoms secondary to a right flank abdominal tumor. An exploratory laparotomy was performed, removing the tumor and applying optimal debulking. The histopathological study reported bilateral ovary adenocarcinoma, as well as metastatic collision tumor of two histological types: well differentiated adenocarcinoma and a mixed malignant mesodermic Müllerian tumor. The patient was treated with adjuvant chemotherapy with poor results (death in 24 months) [15].

Last but not least in 2014 Mukonoweshuro P presented a case of endocervicosis involving axillary lymph nodes. The patient was a case of benign Müllerian inclusions of mucinous endocervical type (endocervicosis) coexistent with metastatic breast-infiltrating ductal carcinoma in 2 axillary lymph nodes. The inclusions exhibited diffuse positive staining with CK7, PAX8, CA125, and estrogen receptor and were WT1 negative [16].

Bilateral prophylactic salpingo-oophorectomy is a very promising procedure offering the benefits of minimizing the risk of ovarian cancer in patients with mutant base. Recent studies demonstrated the benefits of single-port prophylactic laparoscopic bilateral salpingo-oophorectomy versus conventional multiport access in high-risk patients for ovarian cancer. Comparing the results, single-port incision was offering the benefits of less postoperative pain, best esthetic outcome, and faster and easier extraction of the surgical specimens [17].

The patient of the present case was considered as having a stage IV tumor according to the FIGO classification system because of the radiological findings on her CT scan of the thorax and brain even though they were not histologically confirmed as metastases. This confirmation came with the results of the biopsies of the mouth lesions, which were considered metastases from the known sarcoma.

Despite the fact that sarcomas have a long-term metastatic potential, to our knowledge this is the first case of Müllerian adenosarcoma presenting with such extraperitoneal metastases.
